# Brachial Artery Thrombosis following Multiple Wasp Bites

**DOI:** 10.1155/2021/6631126

**Published:** 2021-02-22

**Authors:** K. U. I. S. Gunathilake, M. I. M. Rifath, D. D. Ayeshmantha, J. K. K. N. Jayasinghe, A. Marasinghe

**Affiliations:** ^1^Post Graduate Institute of Medicine, University of Colombo, Colombo, Sri Lanka; ^2^Teaching Hospital Anuradhapura, Anuradhapura, Sri Lanka

## Abstract

Wasp bites can give rise to multiple clinical manifestations ranging from local reactions to multisystem involvement. Stroke and myocardial infarctions following wasp envenomation are reported in the literature. We describe a rare case of brachial artery thrombosis following multiple wasp bites.

## 1. Introduction

Wasp bites are common encounters in general medical wards in Sri Lanka [1]. Wasps, categorised under insect order Hymenoptera which comes under phylum Arthropoda, are the third largest of all insect orders [2]. Despite the benefits of wasps, wasp sting may lead to serious health issues ranging from simple local reactions to life-threatening systemic complications including anaphylaxis and arterial thrombosis. Vascular thrombosis is a rare manifestation of wasp venom; thus, only few cases have been reported in the literature. The pathophysiology of vascular thrombosis may include vasoactive inflammatory and thrombogenic properties of venom, as well as vascular spasm caused by the venom [2–4].

The reported cases of vascular thrombosis include cerebral artery thrombosis and ischaemic strokes, descending aortic thrombosis, coronary artery thrombosis, and myocardial infarctions but never a brachial artery thrombosis to the best of our knowledge [3, 5–7].

## 2. Case Report

**Figure 1:**
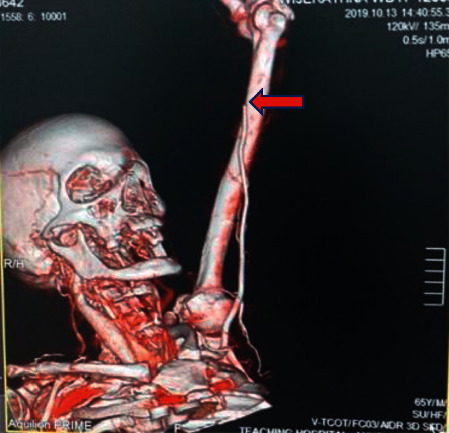
CT angiogram showing the left brachial artery occlusion (red arrow).

**Figure 2:**
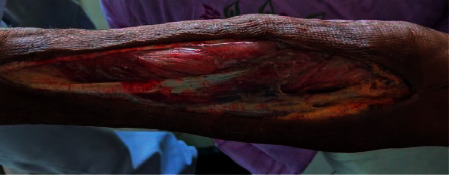
Left arm following fasciotomy.

A 65-year-old previously healthy male from Anuradhapura, north-central part of Sri Lanka, presented to the general medical ward following multiple wasp bites over the head and upper trunk. On admission, he had mild local pain at sting sites but was haemodynamically stable. He developed angioedema of the lips and tongue for which IM adrenaline was given at the emergency treatment unit. Later, he was transferred to the general medical ward for observation. About 8 hours following the bite, he developed severe pain on the left upper limb. On examination, the limb was cold to touch with feeble radial and brachial artery pulses, and finger saturation was not detectable on air. Immediately, vascular surgical opinion was sought, and an IV heparin infusion was started. His CT angiogram revealed long-segment brachial artery thrombosis from the midhumerus level on the left side (Figure 1). Thrombectomy was performed on the same day and fasciotomy the next day to salvage the limb (Figure 2).

Furthermore, he was investigated for a cause for this arterial thrombosis including complete blood count, liver and renal functions, fasting blood sugar, lipid profile, ECG, 2D echo, and clotting profile with thrombophilia screening, all of which yielded normal results.

With the temporal association of events in this previously healthy male in the absence of other risk factors for arterial thrombosis, it was concluded that the brachial artery thrombosis was due to wasp sting.

## 3. Discussion

Wasp bites commonly cause simple allergic reactions, whereas systemic complications including anaphylaxis and arterial thrombosis are rare. Other systemic complications of wasp bite include myasthenia gravis, peripheral neuritis, Guillain–Barre syndrome, diffuse alveolar haemorrhage, acute renal failure, thrombocytopenic purpura, and vasculitis [4, 8]. Wasp venom mainly contains three categories of compounds: (A) high-molecular-weight proteins including phospholipases, hyaluronidases, and antigen V, (B) low-molecular-weight peptides including mastoparans, wasp kinins, and chemotactic peptides, and (C) bioactive molecules including histamine, serotonin, catecholamines, acetylcholine, and tyramine [2].

Large molecules often cause allergic reactions, while smaller compounds in larger quantities lead to systemic reactions. Vasoactive substances cause vascular spasm, vascular inflammation, and thrombosis [2].

Exact pathophysiology of wasp venom-induced arterial thrombosis is yet to be described. It may be multifactorial. There had been several case reports where myocardial infarction was attributed to hypersensitivity, vasospasm, or vascular inflammation following wasp bite [3, 4, 9, 10]. Similarly, stroke either due to hypotension following anaphylaxis or direct vascular toxicity has been reported [6, 11, 12]. Leukotrienes and thromboxane in venom cause platelet aggregation and thrombosis, whereas phospholipases trigger an IgE-mediated reaction cascade leading to mast cell activation and synthesis of a number of inflammatory mediators [6]. Direct toxic effect of wasp venom compounds and vascular inflammation may also play a role. Vasospasm and blood cell aggregation followed by thrombosis is another possible mechanism. Although adrenaline is a possible culprit for vasospasm, in the brachial artery which is a large artery, vasospasm is unlikely to be the cause for thrombosis. It is most likely that thrombosis was a result of combined effects of vascular inflammation and platelet aggregation due to wasp venom.

## 4. Conclusion

Physicians encounter a large number of wasp bites in general medical wards. Although rare, vascular thrombosis is an important clinical outcome of wasp stings as the disability caused by such complications is severe and could have long-term effects on the quality of life of the patient. Prompt investigation and management should be implemented without a delay in such a clinical scenario.

## References

[1] Kularatne S. A. M., Shahmy S., Rathnayake S. S., Dawson A. H. (2018). Clinico-epidemiology of arthropod stings and bites in primary hospitals of North Western province of Sri Lanka. *Clinical Toxicology*.

[2] Dongol Y., Dhananjaya B. L., Shrestha R. K., Aryal G. (2014). Pharmacological and immunological properties of wasp venom. *Pharmacology and Therapeutics*.

[3] Wagdi P., Mehan V. K., Bürgi H., Salzmann C. (1994). Acute myocardial infarction after wasp stings in a patient with normal coronary arteries. *American Heart Journal*.

[4] Greif M., Pohl T., Oversohl N., Reithmann C., Steinbeck G., Becker A. (2009). Acute stent thrombosis in a sirolimus eluting stent after wasp sting causing acute myocardial infarction: a case report. *Cases Journal*.

[5] Chen D. M., Lee P. T., Chou K. J. (2004). Descending aortic thrombosis and cerebral infarction after massive wasp stings. *American Journal of Medicine*.

[6] Dalugama C., Gawarammana I. B. (2018). Ischemic stroke following a wasp sting-a rare complication: a case report. *Journal of Medical Case Reports*.

[7] Chen D.-M., Lee P.-T., Chou K.-J. (2004). Descending aortic thrombosis and cerebral infarction after massive wasp stings. *The American Journal of Medicine*.

[8] Das R. N., Mukherjee K. (2008). Asian wasp envenomation and acute renal failure: a report of two cases. *McGill Journal of Medicine: MJM: An International Forum for the Advancement of Medical Sciences by Students*.

[9] Dechyapirom W., Cevik C., Nugent K. (2011). Concurrent acute coronary syndrome and ischemic stroke following multiple bee stings. *International Journal of Cardiology*.

[10] Cvetkovic-Matic D., Ašanin M., Matic D. (2009). Acute myocardial infarction following a hornet sting. *Vojnosanitetski Pregled*.

[11] Varuni K., Sivansuthan S., Joseph Piratheepan G., Gajanthan R. (2018). A case report on unusual cause of young ischemic cerebrovascular accident: a rare complication of honey bee stings. *Jaffna Medical Journal*.

[12] Crawley F., Schon F., Brown M. M. (1999). Cerebral infarction: a rare complication of wasp sting. *Journal of Neurology Neurosurgery and Psychiatry*.

